# Defining the Minimal Important Difference for the Visual Analogue Scale Assessing Dyspnea in Patients with Malignant Pleural Effusions

**DOI:** 10.1371/journal.pone.0123798

**Published:** 2015-04-15

**Authors:** Eleanor K. Mishra, John P. Corcoran, Robert J. Hallifax, John Stradling, Nicholas A. Maskell, Najib M. Rahman

**Affiliations:** 1 Oxford Centre for Respiratory Medicine and Oxford Respiratory Trials Unit, Oxford Biomedical Research Centre, Churchill Hospital, Oxford, United Kingdom; 2 University of Bristol, Bristol, United Kingdom; Postgraduate Institute of Medical Education and Research, INDIA

## Abstract

**Background:**

The minimal important difference (MID) is essential for interpreting the results of randomised controlled trials (RCTs). Despite a number of RCTs in patients with malignant pleural effusions (MPEs) which use the visual analogue scale for dyspnea (VASD) as an outcome measure, the MID has not been established.

**Methods:**

Patients with suspected MPE undergoing a pleural procedure recorded their baseline VASD and their post-procedure VASD (24 hours after the pleural drainage), and in parallel assessed their breathlessness on a 7 point Likert scale.

**Findings:**

The mean decrease in VASD in patients with a MPE reporting a ‘small but just worthwhile decrease’ in their dyspnea (i.e. equivalent to the MID) was 19mm (95% CI 14-24mm). The mean drainage volume required to produce a change in VASD of 19mm was 760ml.

**Interpretation:**

The mean MID for the VASD in patients with a MPE undergoing a pleural procedure is 19mm (95% CI 14-24mm). Thus choosing an improvement of 19mm in the VASD would be justifiable in the design and analysis of future MPE studies.

## Introduction

Malignant pleural effusions (MPEs), defined as the accumulation of fluid in the pleural space secondary to cancer, cause disabling breathlessness and impair quality of life in over 1 million people worldwide per year[1, 2]. Currently recruiting and recently reported randomised controlled trials (RCTs) use the visual analogue scale for dyspnea (VASD) to assess mean daily breathlessness in patients with malignant pleural effusions (MPEs) (ISRCTN12852177, ISRCTN4784593, ISRCTN73255764) in order to provide evidence for the optimal method of symptom palliation[3]. Interpretation of the results of these RCTs requires determination of the minimal important difference (MID) for the VASD.

The VASD is a patient reported outcome measure, consisting of a 100mm horizontal line labelled at 0mm with ‘Not breathless at all’ and at 100mm with ‘Worst possible breathlessness’. Participants answer the question ‘On average how breathless have you felt in the last 24 hours?’ by marking the line at a point representing their dyspnea intensity. The score is calculated by measuring from 0mm to the mark. The precise appearance of the VAS varies, with the line drawn either vertically or horizontally and with or without subdivisions. These variations do not affect the precision of measurement of change over time by the VAS[4]. In this work, a horizontal, unmarked VAS was used.

The MID is the smallest difference in score that patients perceive as worthwhile, and that would lead them to consider a change in management[5]. It encompasses a trade off between the benefits and disadvantages of a treatment and as such is an important patient-related outcome measure. This form of outcome measure is particularly important in conditions such as MPE where the treatment intent is palliative. The MID depends on context, such as underlying disease and treatment[6]. The optimal method for determining the MID is an anchor approach which compares changes between the tool of interest and an anchor for which the MID is known, such as a Likert scale[6]. An alternative is an opinion approach (i.e. ask patients what they consider to be the MID). Statistical approaches, such as the effect size (ES) index and empirical rule effect size (ERES) infer the MID based on the distribution of data in the sample. The ES is defined as the difference in means divided by the standard deviation. A change in score with an ES of 0.33 is considered to approximate the MID [7]. The ERES assumes that scores are normally distributed, with a mean score of half the maximum value and the range of the score encompasses six SDs, so the MID for any 100 point tool is estimated to be 8.4[8].

Two previous studies have estimated the MID for the VASD using a 5-point Likert scale in acute dyspnea in patients attending the emergency department and found it to be 22mm (95% CI 11–34mm) in patients with an exacerbation of asthma and 21mm (95% CI 12–30mm) in patients with decompensated heart failure[9, 10].

A further aim of this work was to investigate the effect of the magnitude of the patient’s initial symptoms on the MID. Some researchers have found a relationship between baseline score and MID e.g. Ander et al. found that patients with a low initial level of dyspnoea (initial VASD 0–50mm) considered a mean change in VAS of 5.7mm (95% confidence interval -3.6–15.0mm) to represent either ‘a little less difficulty breathing’ or ‘a little more difficulty breathing’ whereas for patients with a high initial VAS (51–100mm) considered a mean change of 30.9mm (95% CI 19.1–42.6mm) to represent this [10]. However, this apparent dependency may be due to mathematical coupling, rather than a true dependency. Mathematical coupling occurs ‘when one variable directly or indirectly contains the whole or part of another’[11]. Therefore Oldham’s method will be used to assess if there is a true relationship between baseline VASD and MID. Oldham demonstrated that for two series of independent random numbers *x* and *y* with the same standard deviation, there is a strong correlation of approximately 0.71 between *x* (i.e. the baseline measurement) and *x*-*y* (i.e. the change in measurement). This has led to some researchers inappropriately concluding that there is a correlation between baseline score and change in score and therefore that the MID is dependent on baseline score. Oldham demonstrated that calculating the correlation between *x*-*y* and (*x+*y)/2 lead to a true assessment of the correlation between the variables. This is therefore an unbiased test of the relationship between baseline VASD and change in VASD.

The primary aim of this study was to determine the MID for the VASD in patients with a MPE using a Likert scale as an anchor. The MID was defined as the mean decrease in VASD in subjects experiencing a ‘small but just worthwhile’ improvement in dyspnea. Secondary outcomes were an opinion based estimate of the MID, statistical estimate of the MID, and the relationship between change in VASD and volume of pleural fluid drained.

## Methods

This study was discussed with the chair of an Oxford Research Ethics Committee. The data analysed was collected as part of our standard clinical practice to assess response to pleural fluid drainage and therefore we were advised that this assessment could be considered an audit of practice, and thus research ethics approval and written informed consent was not required.

**Fig 1:**
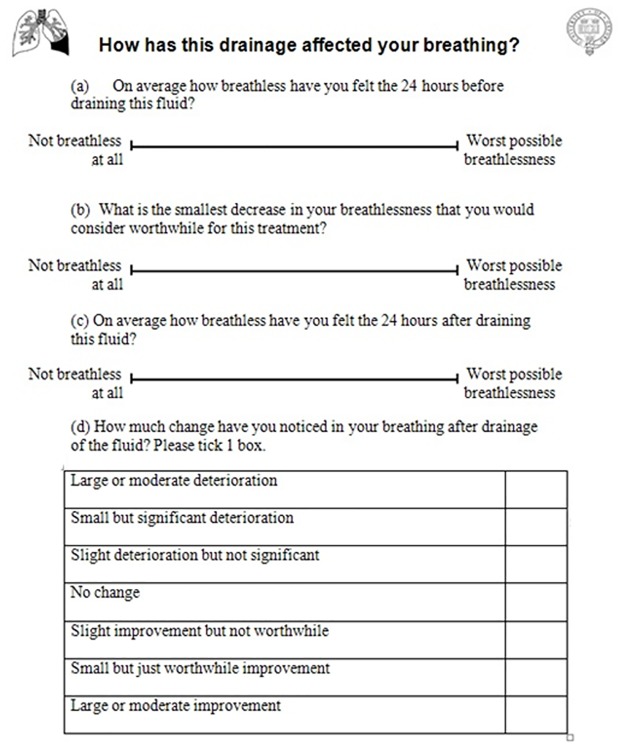
Study questionnaire. Questionnaire used to assess dyspnea before and after a pleural procedure.

The study consisted of an analysis of data collected in our clinical practice wherein a questionnaire (Fig 1) is administered to dyspnoeic adult patients with a confirmed or suspected MPE undergoing a pleural procedure (diagnostic or therapeutic aspiration, chest drain insertion, indwelling pleural catheter (IPC) insertion and/or drainage or local anaesthetic thoracoscopy(LAT)). Prior to the procedure, clinical staff explained the procedure to the patient, discussed risks and benefits and obtained informed consent for the procedure. Immediately prior to the procedure, patients recorded their baseline VASD and estimated the greatest post-procedure VASD that the patient would consider to be “worthwhile” for the procedure they were about to have (greatest worthwhile VASD). The questionnaire was not administered to patients with visual impairment. Patient information was anonymized and de-identified prior to analysis.

Approximately 24 hours after the procedure, patients completed their post-procedure VASD and a 7-point Likert scale with the following options: large or moderate improvement; small but just worthwhile improvement (equivalent to the MID); slight improvement but not worthwhile; no change; slight deterioration but not significant; small but significant deterioration; and large or moderate deterioration. Patients were able to see their baseline VASD when assessing their post-procedure VASD, as there is evidence that permitting subjects see their previous responses increases the validity of patient-reported outcomes[12].

Date of birth, gender, diagnosis, procedure performed and volume of fluid drained in 24 hours were recorded (S1 Data Set).

All patients on whom data were available were included in the analyses. All analyses were pre-planned prior to review of any data. SPSS version 20.0.0 (*IBM*, *New York*, *USA*) was used. Patients were categorised based on response to the Likert scale described above. After analysis of the distribution of data, the mean decrease in VASD following the procedure was calculated for each group. Additionally, 95% CIs were calculated. Patient opinion estimation of the MID was calculated by subtracting the greatest worthwhile VASD from the baseline VASD. The decrease in VASD corresponding to an effect size of 0.33 was calculated as baseline SD divided by 3.

## Results

A total of 114/123 (93%) questionnaires were returned January—December 2012. For the primary outcome, data was available in 106/114 (93%) questionnaires. For demographics, data was available in 108/114 (95%) questionnaires. Mean age was 70 years. Sixty (56%) patients were female. Ninety-two patients (81%) had a final diagnosis of MPE. Diagnoses and types of pleural procedure are summarised in Tables 1 and 2.

**Table 1:** Summary of final diagnoses.

Diagnosis	Number of patients (%)
**Breast cancer**	38 (34)
**Mesothelioma**	24 (21)
**Non-small cell lung cancer**	17 (15)
**Ovarian cancer**	4 (3.5)
**Leiomyosarcoma**	2 (1.8)
**Colorectal cancer**	3 (2.6)
**Other malignant effusions**	4 (3.5)
**Benign pleural effusion**	16 (14)
**Data missing**	6 (5.3)

**Table 2:** Number of different pleural procedures.

Procedure	Number of patients (%)
**Intercostal drain**	13 (11)
**Diagnostic aspiration**	12 (11)
**IPC drainage**	1 (0.88)
**IPC insertion and drainage**	10 (8.8)
**Therapeutic aspiration**	40 (8.8)
**Thoracoscopy**	31 (27)
**Data missing**	7 (6.1)

Mean baseline VASD was 57mm. Mean decrease in VASD was 30mm. Mean volume of pleural fluid drained within 24 hours was 1200ml. All continuous data were normally distributed.

**Fig 2:**
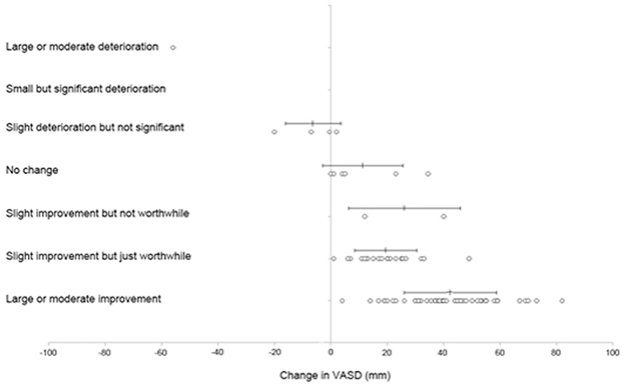
Decrease in visual analogue scale for dyspnea based on response on Likert scale after procedure. Circles represent individual data points with mean and standard deviation marked above. Positive change in VASD represents improvement in breathlessness.

The mean decrease in VASD for patients with a final diagnosis of a MPE who experienced a ‘small but just worthwhile improvement’ was 19mm (SD 11mm, 95% CI 14–24mm) (Table 3, Fig 2). Including patients with benign effusions, the equivalent value was 21mm (95% CI 15–26mm).

**Table 3:** Summary of decrease in VASD categorised by response on Likert scale.

Likert scale	No. of patients	Decrease in mean VASD (mm)	95% CI (mm)
**Large or moderate improvement**	50	42	38–47
**Small but just worthwhile improvement**	20	19	14–24
**Slight improvement but not worthwhile**	2	26	-151-203
**No change**	6	11	-3.6–26
**Slight deterioration but not significant**	4	-6.4	-22–9
**Small but significant deterioration**	0	-	-
**Large or moderate deterioration**	1	-56	-

The minimum mean decrease in VASD that patients considered to be worthwhile for the treatment they were undergoing was 24mm (95% CI 21 – 27mm). Of note, this concept was difficult for patients to grasp, and common responses to this question were ‘Any improvement would be worthwhile’ and ‘This is where I want to be’ (indicating 0mm/’not breathless at all’).

**Fig 3:**
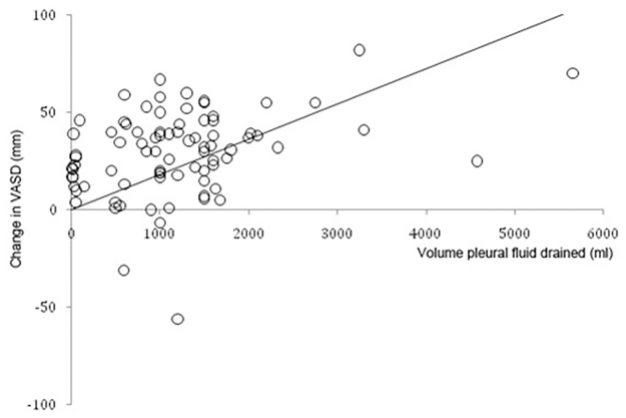
Relationship between volume of fluid drained and change in visual analogue scale for dyspnea. Circles represent individual data points (change in visual analogue scale and volume of pleural fluid drained) following pleural procedures. Solid line represents the line of best fit going through zero.

Linear regression analysis demonstrated that baseline VASD (β = 0.39, p<0.001) and volume drained (β = 0.24, p = 0.02) were significant predictors of decrease in VASD. Age, sex, diagnosis and procedure performed were not significant predictors. The overall model fit was R^2^ = 0.36. Based on these results, the mean volume of fluid to drain in order to produce a mean change in VASD of 19mm (i.e. to produce a change in symptoms equivalent to the MID) was 760ml (Fig 3).

Overall, 70/83 (84%) of patients had a worthwhile or greater benefit.

**Fig 4:**
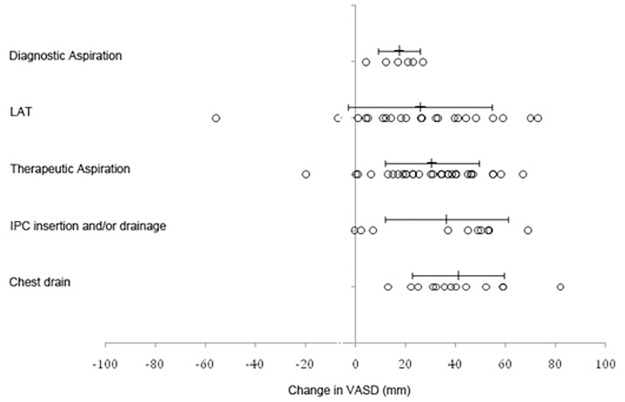
Mean decrease in visual analogue scale for dyspnea based on different procedures. Circles represent individual data points with mean and standard deviation marked above. Positive change in VASD represents improvement in breathlessness.

Mean (SD) decrease in VASD for the different procedures were: chest drain 41mm (18); IPC insertion and drainage 41mm (22); therapeutic aspiration 31mm (18); diagnostic aspiration 19mm (10); and LAT 24mm (30) (Fig 4). ANOVA on these values for these five procedures showed that there were significant differences between these results (*F*(4, 94) = 2.7, *p* = 0.04).

There was a significant correlation between the initial VASD and the decrease in VASD following a pleural procedure (*r* = 0.60, p<0.001). However, using Oldham’s method (i.e. calculating the correlation between change in measurement and mean of baseline and second measurements as described in the introduction) to correct for the problem of mathematical coupling, it was demonstrated that there was no true correlation between initial VASD and decrease in VASD (*r* = 0.13, p = 0.21).

The SD of pre-procedure VASD scores was 23mm, therefore, based on a ES of 0.33, the MID would be 7.7mm.

## Discussion

The primary outcome for this analysis of clinical data was the change in VASD corresponding to a ‘small but just worthwhile improvement’ on the Likert scale in dyspnea in patients with an MPE undergoing a pleural procedure and was found to be 19mm (95% CI 14 – 24mm). This value is similar to values found by previous studies which have assessed the VASD in a similar way, despite the fact that these studies assessed the MID in different groups of patients and different treatments (patients with heart failure treated with diuretics and asthmatics treated with salbutamol and steroids) and in a different setting (emergency department)[9, 10]. This value of 19mm (95% CI 14 – 24mm) is greater than the value that previous experts have assumed the MID to be (10mm)[13, 14]. The data here suggest that clinicians assume smaller changes in symptoms on the VASD to be worthwhile compared to patients’ views. It is similar to the value estimated by asking patients’ opinion on what they would consider to be the greatest worthwhile post-procedure VASD (24mm, 95% CI 21–27mm).

Distributional approaches to determining the MID use statistical methods based on the variation within the sample. These approaches have been devised by looking at values for the MID which have been determined by other methods and attempting to find a common statistical formula which can generate similar values. It should be noted that there is no theory underlying the derivation of these statistical formulae. The values calculated in this study using distributional approaches were lower than those calculated using anchor or opinion methods (ES estimating it at 7.7mm and ERES estimating it at 8.4mm). The ES index may underestimate the MID for the VASD because an effect size of 0.5 is too low an effect size to approximate the MID for procedures where there is significant inconvenience and risk. The ERES is based on assumptions which are invalid in this data set, specifically assuming that the mean is 50mm and the SD is 16.7mm. Distributional approaches for determining the MID have previously been criticised for demonstrating a clinically significant and meaningful change, but not a minimal one[6]. In contrast, this work found distributional approaches to underestimate the MID. This difference may be because pleural interventions involve significant inconvenience, pain and risk compared to the interventions that were used to determine distributional approaches. This analysis demonstrated the weakness of distributional approaches to estimating the MID—these approaches are insensitive to factors known to influence the MID, such as treatment and underlying disease.

Our results showed that baseline VASD and volume drained were significant predictors of decrease in VASD, suggesting that drainage of 760ml of fluid would improve dyspnea by the MID. However, the weak correlation between these variables means this data should not be used to guide treatment of individual patients. Other factors, such as diaphragmatic inversion, underlying diagnoses and reaction to pleurodesis are likely to affect dyspnea relief.

These results show that different pleural procedures relieve dyspnoea by significantly different amounts which is unsurprising. However, the numbers of some procedures are small and so the absolute values and results of the ANOVA should be interpreted with caution. It is unclear whether these differences are due only to the volume of fluid drained or whether other factors also affect this. The sample size is too small to distinguish between these possibilities.

Although not the aim of this analysis, this work validates the use of the VASD in patients with MPE. Patients found it acceptable and easy to understand and complete. The VASD is responsive to pleural interventions and correlates with volume of pleural fluid drained, demonstrating construct validity. It also highlights the effectiveness of pleural procedures at relieving dyspnea, at least in the first 24 hours following the procedure, with 84% of patients experiencing a worthwhile or greater improvement in dyspnea.

When completing the questionnaire, patients were able to see their baseline rating when completing the post-procedure VASD because there is evidence that letting subjects see their previous responses increases the validity of patient-reported outcomes[12].

Statistical analysis using Oldham’s method showed no true correlation between baseline VASD and decrease in VASD, demonstrating that the MID is a contant, regardless of the patient’s baseline breathlessness.

The limitation of this estimate of the MID for the VASD is that it applies specifically to patients with MPE undergoing a pleural procedure. A further limitation is that there were very few patients who experienced a ‘slight improvement but not worthwhile’, no change or a deterioration in their symptoms, making it difficult to differentiate the MID from these categories.

In conclusion, this analysis demonstrated that the MID for the VASD in patients with MPEs undergoing a pleural procedure is 19mm (95% CI 14 – 24mm). This result has interesting implications—similar results in previously published research suggest this value may apply to all hospitalised patients with acute dyspnea. Statistical methods (ES or ERES) were unreliable for determining the MID in this context and demonstrate that these methods are inappropriate for estimating the MID in RCTs. However, patient opinion-based methods may be a reasonable substitute for anchor methods.

## Supporting Information

S1 Data Set Data used to determine the minimal important difference for the visual analogue scale for dyspnea. Complete data set used for this work.
(XLSX)
